# Supplementary material for: Metabolic imbalance limits fermentation in microbes engineered for high-titer ethanol production
Source: mSystems. 2026 Mar 25;11(4):e00074-26. doi: 10.1128/msystems.00074-26 (PMC13098246; doi:10.1128/msystems.00074-26)
Supplement: Supplemental material — Figures S1-S7 and descriptions of Files S1-S5. [file msystems.00074-26-s0006.docx]

## SUPPLEMENTARY MATERIALS


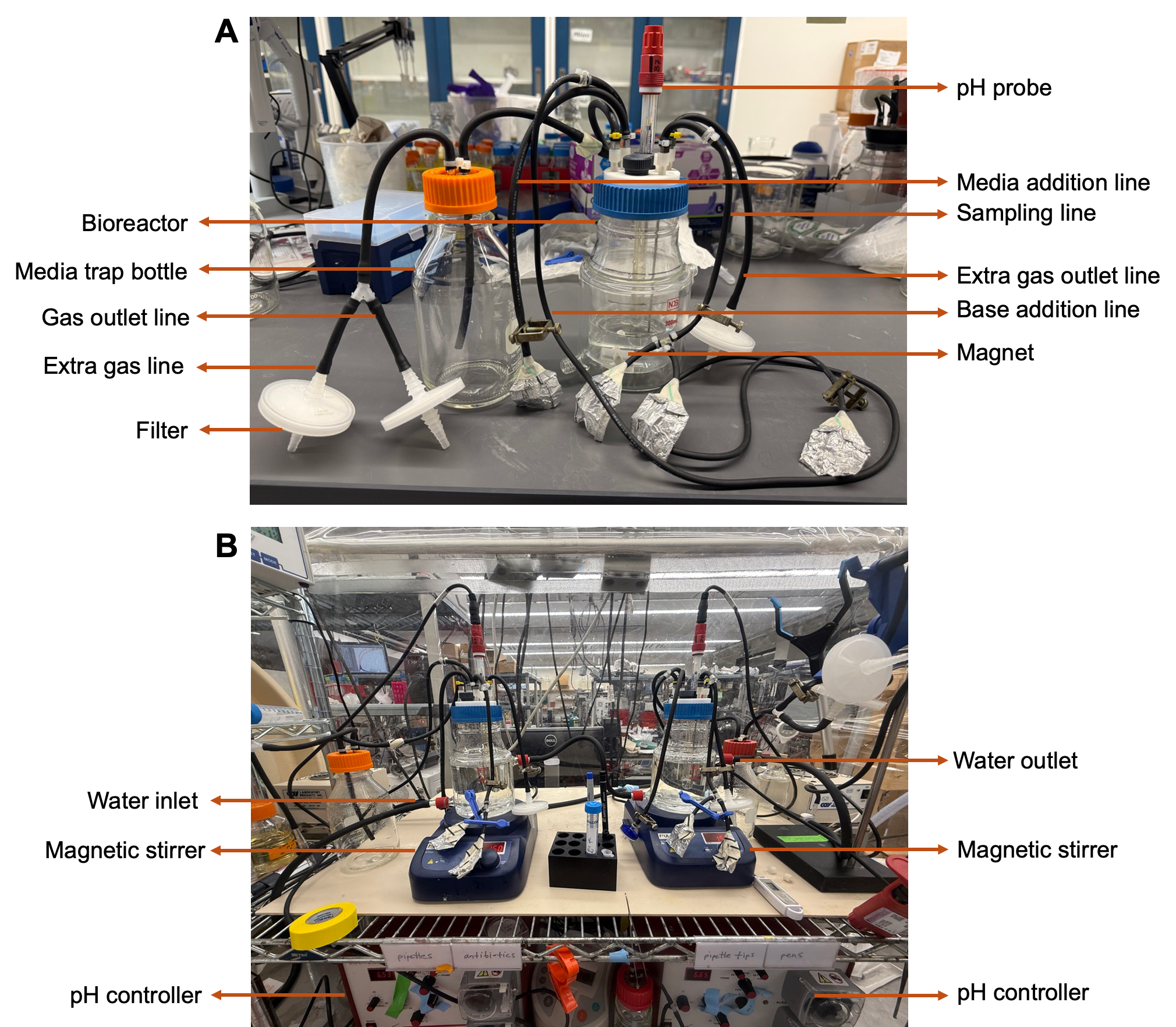


**Figure S1: Bioreactor setup used for the fermentations. A. Bioreactor setup before autoclaving.** The glass reactor was obtained from NDS Glass (Vineland, New Jersey). The media addition line allows for the introduction of media components, while the sampling line facilitates culture sample collection. The gas outlet line enables gas release, and the base addition line is used to pump 4 M KOH into the reactor. Two additional gas lines serve as backups in case the primary gas line becomes obstructed during fermentation. The media trap bottle prevents stray liquid in the gas line from obstructing the filter. A magnetic stir bar inside the reactor ensures mixing. **B. Bioreactors inside anaerobic glove bag.** Two reactors operate in parallel, positioned above magnetic stirrers. Water inlet and outlet lines, connected to a water jacket on the outside of the reactor, regulate reactor temperature via a water bath located outside the glove bag. The pH controller pumps base into the reactor to maintain the set pH.


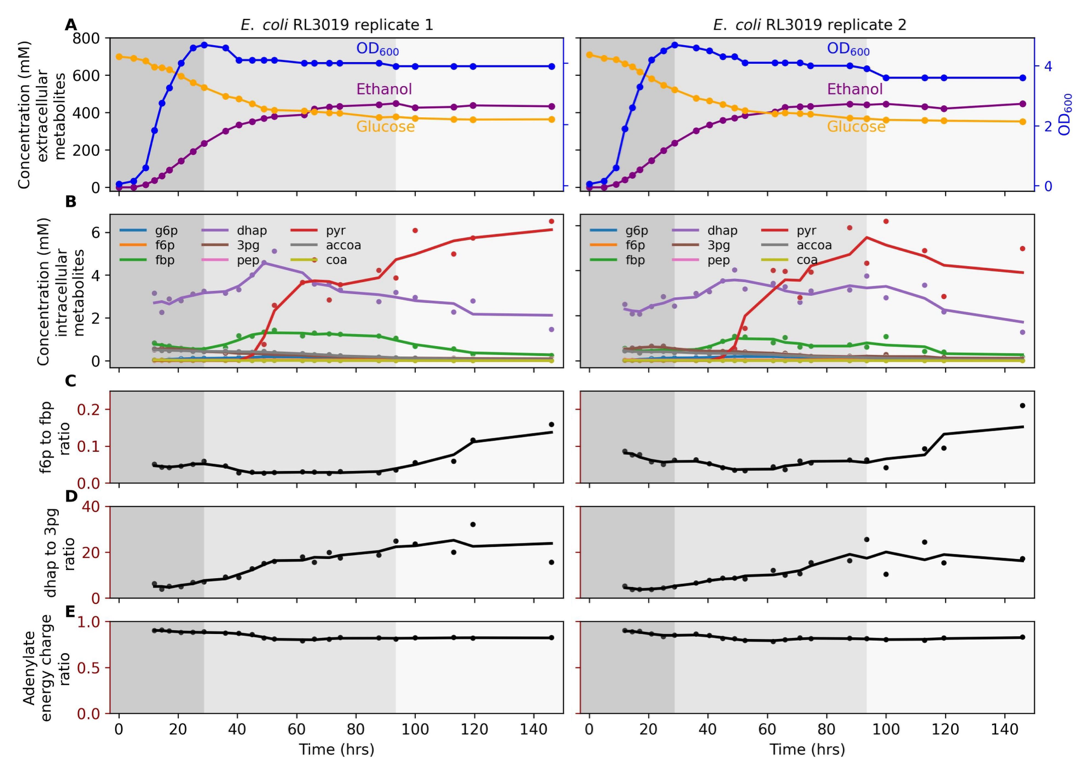


**Figure S2: Comparison of fermentation profile of *E. coli* RL3019 biological duplicates. A) Concentration of extracellular metabolites B) Concentration of intracellular metabolites C) f6p to fbp ratio D) dhap to 3pg ratio E) Adenylate energy charge ratio.** Fermentation was performed with 120 g/L glucose in M9 minimal medium at 37°C with pH-maintained at 6.5 +\- 0.05 by addition of 4 M potassium hydroxide. The shaded background represents different phases of fermentation: dark gray, growth coupled fermentation; medium gray, growth uncoupled fermentation; and light gray, no ethanol production phases. Figure 1 shows replicate 1 (left panel) of this figure. Additional File 1 (extracellular - 2) shows additional extracellular metabolites data for replicate 2. Additional File 1 (intracellular - 2) shows intracellular metabolites concentrations for replicate 2. The abbreviations used in this figure are defined in the list of abbreviations.

**
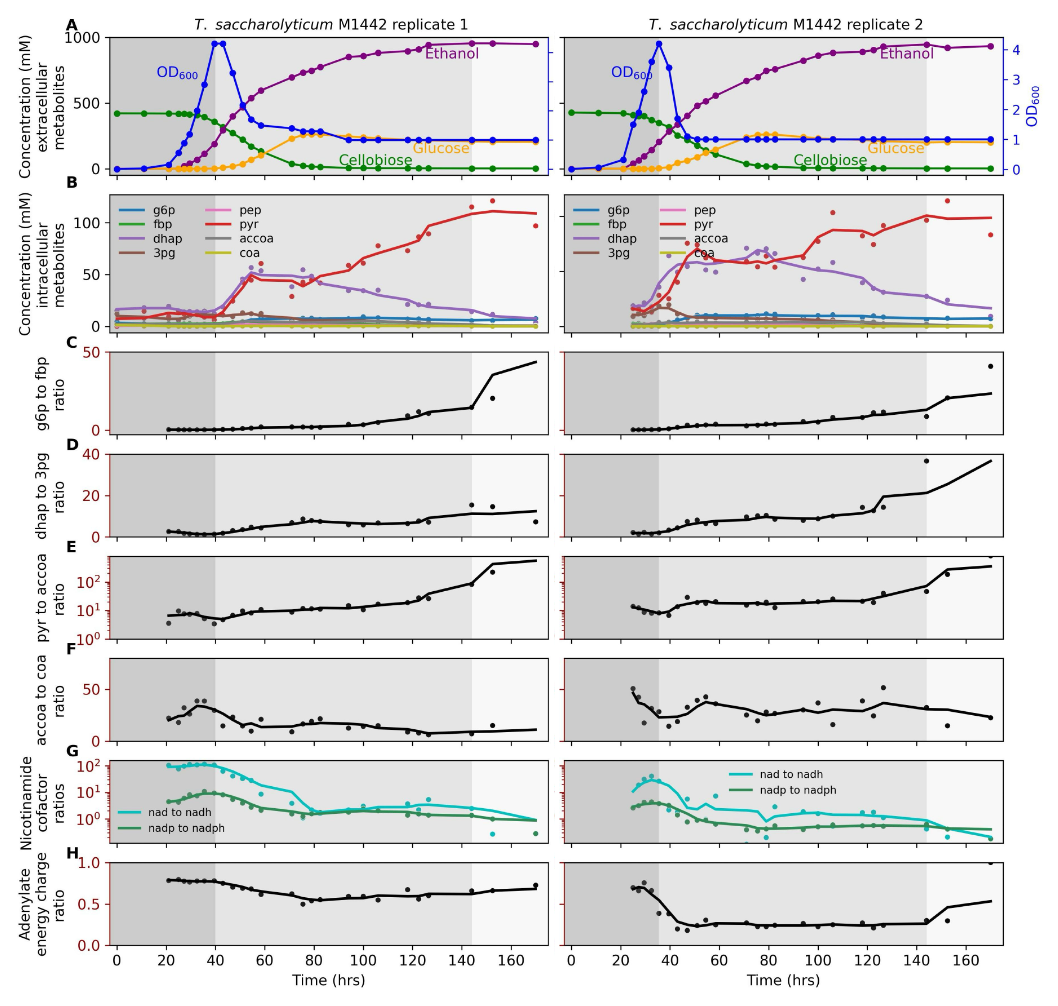
**

**Figure S3:** **Comparison of fermentation profile of *T. saccharolyticum* M1442 biological duplicates. A) Concentration of extracellular metabolites B) Concentration of intracellular metabolites C) g6p to fbp ratio D) dhap to 3pg ratio E) pyr to accoa ratio F) aacoa to coa ratio G) Nicotinamide cofactor ratios H) Adenylate energy charge ratio** Fermentation was performed with 140 g/L cellobiose in MTC-7 medium at 51°C with pH-maintained at 6.0 +\- 0.05 by addition of 4 M potassium hydroxide. The shaded background represents different phases of fermentation: dark gray, growth coupled fermentation; medium gray, growth uncoupled fermentation; and light gray, no ethanol production phases. Figure 2 shows replicate 1 (left panel) of this figure. Additional File 2 (extracellular - 2) shows additional extracellular metabolites data for replicate 2. Additional File 2 (intracellular - 2) shows intracellular metabolites concentration for replicate 2. In subplots B–H, each circle represents an individual measurement, and the line plot represents a trendline, which is smoothed using a rolling average with a window size of 3. The abbreviations used in this figure are defined in the list of abbreviations.


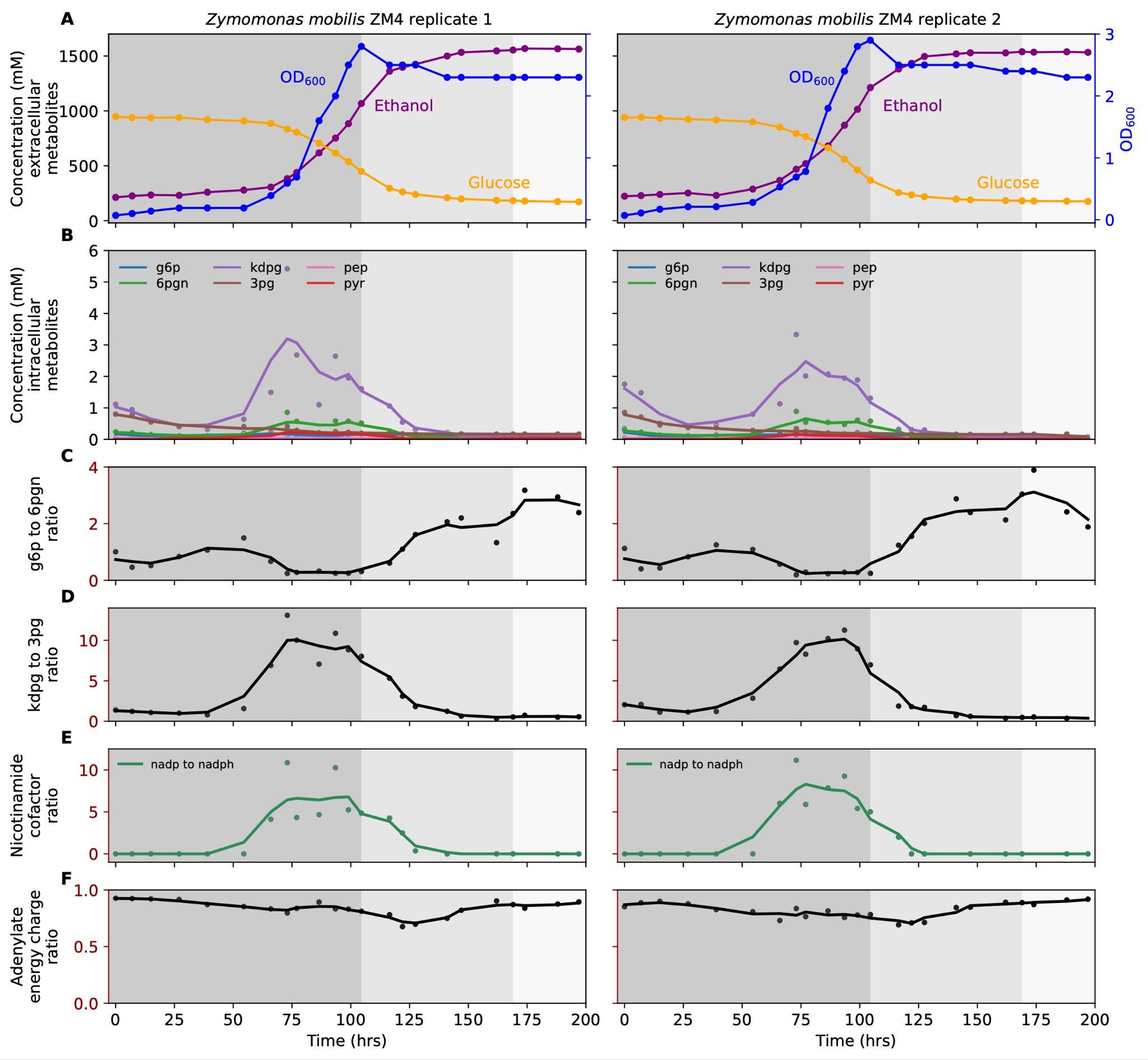


**Figure S4:** **Comparison of fermentation profile of *T. saccharolyticum* M1442 biological duplicates. A) Concentration of extracellular metabolites B) Concentration of intracellular metabolites C) g6p to 6pgn ratio D) kdpg to 3pg ratio E) Nicotinamide cofactor ratio F) Adenylate energy charge ratio** Fermentation was performed with 160 g/L glucose in ZMM-2 medium at 37°C with pH-maintained at 6.0 +\- 0.05 by addition of 4 M potassium hydroxide. The shaded background represents different phases of fermentation: dark gray, growth coupled fermentation; medium gray, growth uncoupled fermentation; and light gray, no ethanol production phases. One representative fermentation profile is shown (n=2). Figure S4 shows a biological duplicate of this experiment. Additional File 3 (extracellular - 2) shows additional extracellular metabolites data for this experiment. Additional File 3 (intracellular - 2) shows intracellular metabolites concentration. In subplots B–H, each circle represents an individual measurement, and the line plot represents a trendline, which is smoothed using a rolling average with a window size of 3. The abbreviations used in this figure are defined in the list of abbreviations.


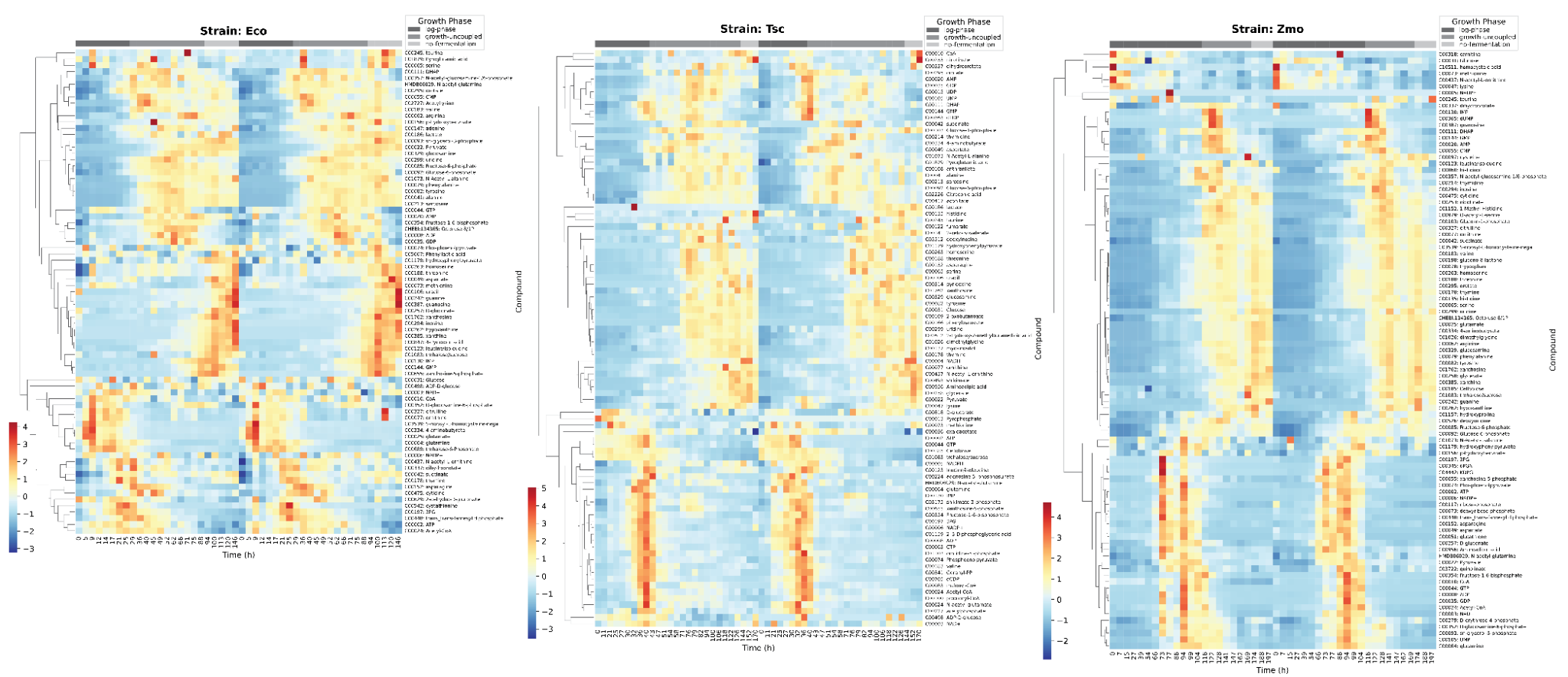


**Figure S5:** **Relative abundance of metabolites during fermentation - all data and replicates.** This figure shows additional replicates and metabolites not shown in Figure 4.

**
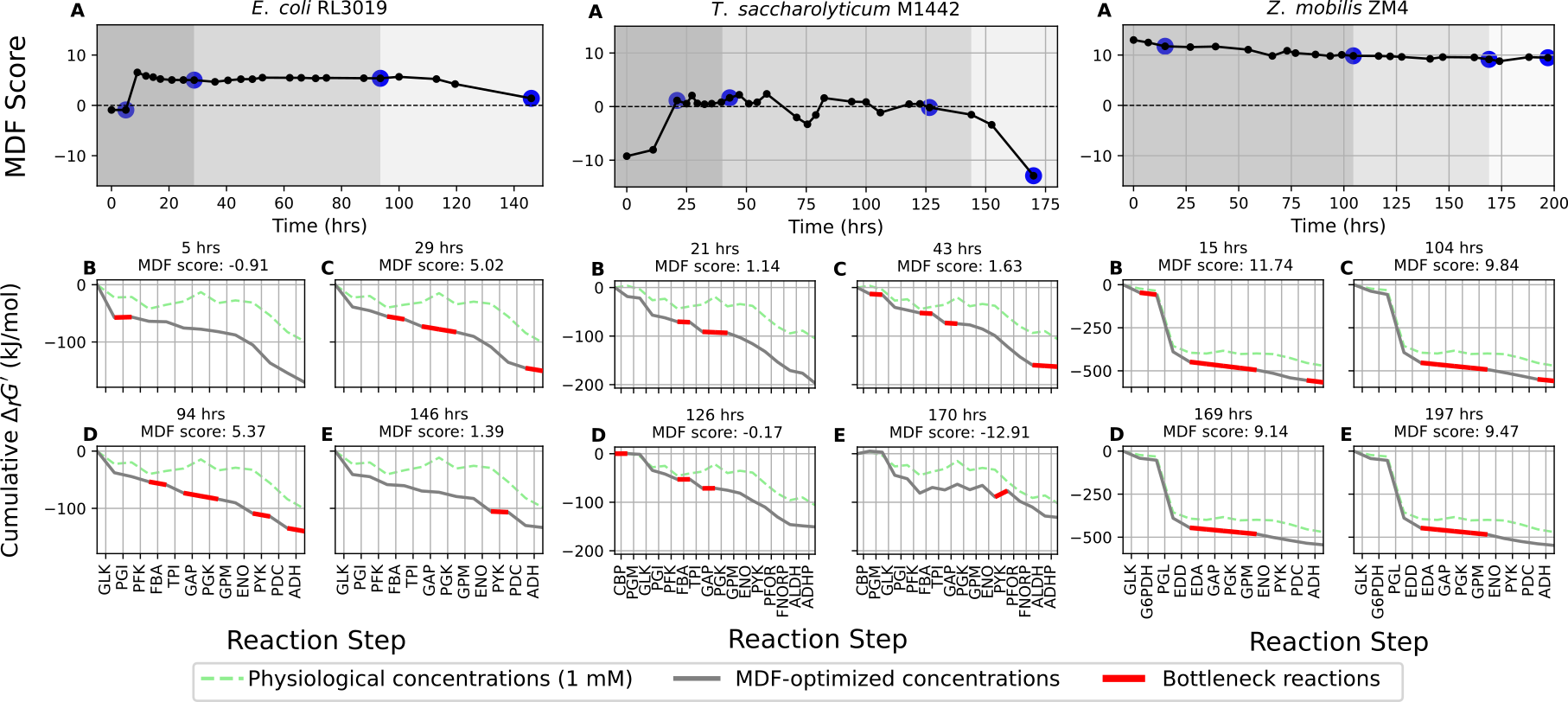
**

**Figure S6: Max-Min Driving Force (MDF) scores during fermentations of *E. coli* RL3019 (left), *T. saccharolyticum* M1442 (middle), and *Z. mobilis* ZM4 (right).** MDF scores were calculated at each time point during the course of fermentation. The blue circles represent the specific time points for which MDF scores are plotted. In the top row of panels, the shaded backgrounds indicate different phases of fermentation: dark gray, growth-coupled fermentation; medium gray, growth-uncoupled fermentation; and light gray, no ethanol production. For each organism, the four lower subplots represent cumulative ∆rGʹ for the blue-circle time points in the MDF score chart. The green dotted line shows cumulative ∆rGʹ when metabolite concentrations are fixed at 1 mM, the gray line shows values based on measured concentrations, and the red line highlights the bottleneck reactions responsible for changes in MDF scores. Biological replicates of these experiments are shown in Figures 6. Abbreviations used in this figure are defined in the list of abbreviations.


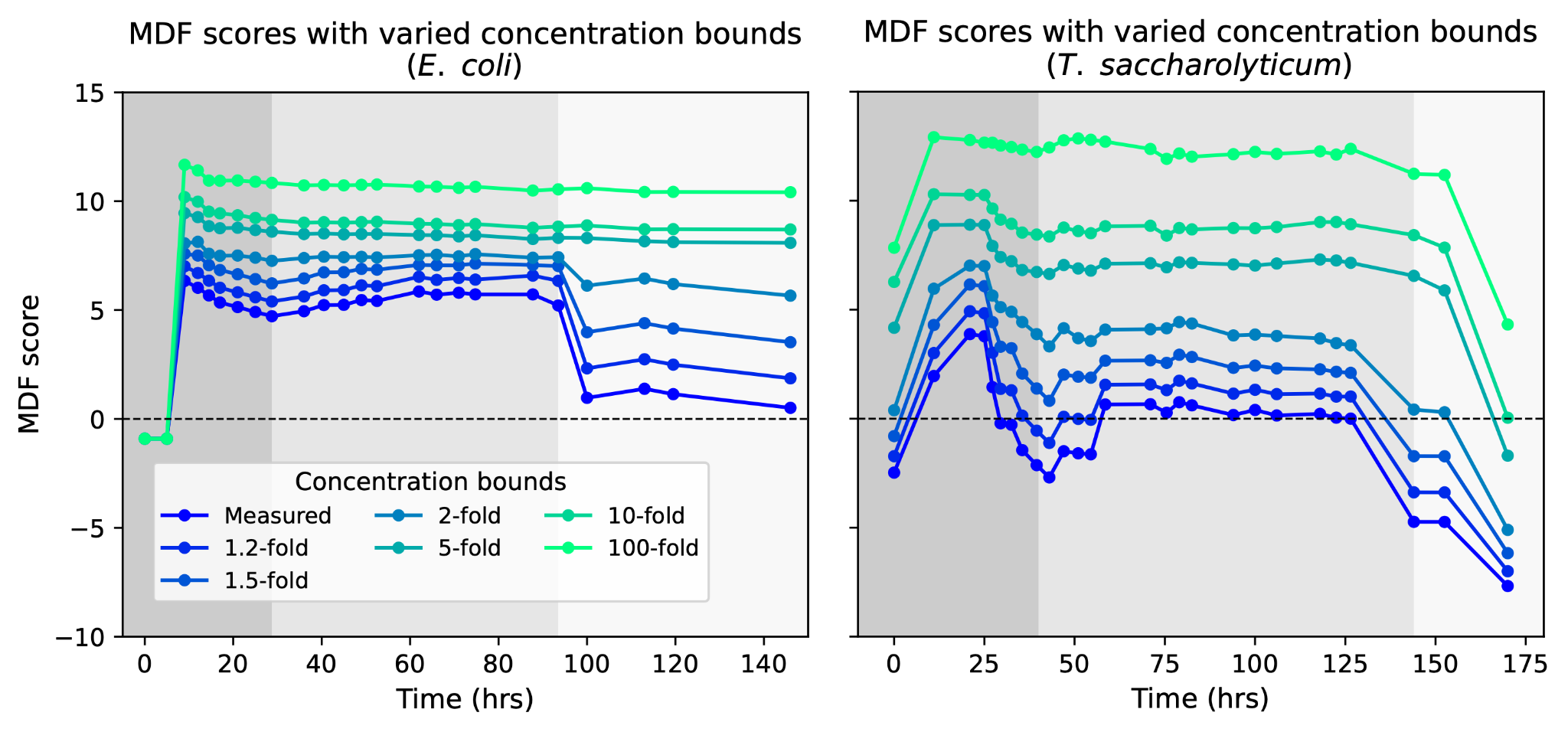


**Figure S7: Change in Max-Min Driving Force (MDF) score by applying bounds to *E. coli* (left panel) and *T. saccharolyticum* (right panel).** The concentration bound represents the allowed lower and upper limit from measured metabolites concentrations. The shaded background represents different phases of fermentation: dark gray, growth coupled fermentation; medium gray, growth uncoupled fermentation; and light gray, no ethanol production phases.

**Additional File 1: Metabolite concentrations for *E. coli*, *T. saccharolyticum*, and *Z. mobilis* during the course of fermentation. All concentrations are reported in millimolar (mM).** Excel sheets are organized by organism, replicate, and sample type, with each sheet containing time-course data. For *E. coli*, eco1_ext and eco1_int correspond to extracellular and intracellular metabolite concentrations for replicate 1, and eco2_ext and eco2_int correspond to replicate 2. For *T. saccharolyticum*, sheets are labeled tsac1_ext, tsac1_int, tsac2_ext, and tsac2_int for replicates 1 and 2. For *Z. mobilis*, sheets are labeled zmm1_ext, zmm1_int, zmm2_ext, and zmm2_int for replicates 1 and 2. The sheet fermentation_all_ext contains fermentation data and replicates for the data shown in Table 2.

**Additional File 2: Stoichiometry of reactions used for MDF analysis.** The excel sheet ‘Reaction’ contains the stoichiometric matrix of the fermentation pathways for *E. coli* RL3019, *T. saccharolyticum* M1442, and *Z. mobilis* ZM4. Each row corresponds to a reaction and lists the associated gene, sub-pathway classification, reaction ID, reaction formula, and flux scaling factor. The stoichiometric matrix specifies the coefficients for each metabolite, with negative values indicating reactants and positive values indicating products. Additional columns indicate the stoichiometry of each reaction in the three microbes. The sheet ‘Compound’ provides metabolite IDs and KEGG identifiers for all compounds included in the network.

**Additional File 3: Raw and Quantified LCMS data.** The excel file contains raw data for metabolites measured by LCMS, as well as absolute quantification for selected metabolites. The columns contain data as follows:

‘compound’: Compound name.

‘compoundId’: KEGG identifier.

‘peakAreaTop’: The relative metabolite abundance based on the average intensity of the top three points of a peak and is the default peak intensity metric used in the Maven software used for LCMS data analysis.

'extract concentration (uM)': Concentration of the metabolite in the metabolite extraction solution. Values are derived from 'peakAreaTop' using a calibration curve with known external standards.

‘Volume of metabolite extraction buffer (mL)': Volume of extraction buffer used to extract intracellular metabolites from the cells.

'Time (hrs)': Timepoint during the fermentation when samples were taken from the bioreactor. ‘OD’: Optical density (OD600) of the culture at the time of harvest.

‘Volume taken from culture (mL)’: Volume of cell culture applied to the filter for metabolite extraction.

'cell_no': Number of cells per mL at OD600 = 1.

'cell volume (mL)': Intracellular volume of a single cell.

'intracellular concentration (mM)': Intracellular concentration of compounds in a cell (see material and methods for detailed calculation).

‘microbe’: Organism: *E. coli* (eco), *T. saccharolyticum* (tsc), or *Z. mobilis* (zmm) .

‘replicate’: Biological replicate number of the fermentation.

**Additional File 4: KEGG metabolite groups.** This file gives information about KEGG IDs, metabolites, and the groupings that were used to generate the metabolite subsets in Figure 4.

**Additional File 5: Figure generation code.** This Jupyter Notebook file provides the computer code used to generate the heatmap plots in Figure 4 and Figure S5. Additional Files 3 and 4 are used as inputs for this data processing.
